# Race and satisfaction in general OB/GYN clinics

**DOI:** 10.1186/1472-6874-5-6

**Published:** 2005-05-12

**Authors:** James E Rohrer, Jon D Lund, Susan Goldfarb

**Affiliations:** 1 Department of Family and Community Medicine, Texas Tech University Health Sciences Center, Amarillo TX, USA; 2 Department of Obstetrics and Gynecology, Texas Tech University Health Sciences Center, Amarillo TX, USA; 3 Department of OB-GYN, Naval Medical Center San Diego, San Diego CA, USA

## Abstract

**Background:**

The purpose of this study was to test the hypothesis that racial differences in satisfaction can be found among OB/GYN patients on a US naval base.

**Methods:**

Cross-sectional surveys assessing satisfaction with services were obtained from 838 patients who were seen in one of the two general OB/GYN clinics (455 in the base hospital clinic and 391 in a satellite clinic). Multiple logistic regression analysis was used to identify subgroups of patients who were not very satisfied with care received.

**Results:**

When the patients seen in the base hospital were analyzed separately, Asian respondents had significantly lower odds of being very satisfied relative to non-Hispanic white respondents (AOR = .33, p < .01).

**Conclusion:**

Asian patients may be less satisfied than others when treated at a larger facility. Patients treated at a satellite clinic tended to be more satisfied than patients seen at the base hospital.

## Background

Patient satisfaction has become widely regarded as an important performance indicator for health systems [1]. Patients are the best judge of those aspects of care that matter the most to them [2]. Therefore, patient-centered health systems seek to achieve high levels of patient satisfaction. However, fewer studies reported in the OB/GYN literature focus on satisfaction than might be expected. Hospital maternity care has been assessed using patient satisfaction [3,4] and patient satisfaction has been used to compare OB/GYN to other providers or specialties [5,6]. An increasing number of studies have evaluated the benefits of particular procedures using patient satisfaction as an outcome measure [7-13].

This report adds to the limited fund of information about the determinants of patient satisfaction among OB/GYN clinic patients. Studies of other types of medical care have found racial disparities in satisfaction [14-16] and better satisfaction with midlevel providers than with MDs in either pediatrics or adult medicine clinics [17]. Organizational issues appear to be important as well: women seen in women's clinics are more satisfied than those attending mixed-gender clinics [18], patients seeing providers of the same race are more satisfied than those seeing a physician of a different race [19], free-standing clinics may be scored higher than hospital based clinics by patients [1], and clinics achieving a higher level of provider continuity may also attain higher levels of patient satisfaction, provided that patients may change providers when they so desire [20]. Whether any of these relationships hold true for OB/GYN care is not known. Racial disparities in health status, access to care and quality have been demonstrated in many venues; these, therefore, will be the focus of this report.

The purpose of this study was to test the hypothesis that racial differences in satisfaction can be found among patients seen in general OB/GYN clinics operated by the Naval Medical Center in San Diego. By using just one health system, most organizational characteristics were ruled out as possible causes of differences in satisfaction. In addition, cost to the patient was not a factor since all care was free to the user. In effect, the military medical care system in a single city provides a natural laboratory for assessing the effects of race on satisfaction with minimal confounding from other variables such as poverty and variations in the availability of providers.

## Methods

Over a two-month period, a convenience sample of 1544 women receiving OB/GYN services at a large military hospital completed a two-page questionnaire. The survey was approved by the Institutional Review Board. No written consents were required.

The questionnaire was formatted on a two-sided standard automated data form, which was distributed randomly to patients receiving OB/GYN care at all department clinics, as well as to antepartum and post-partum inpatients. With receipt of the survey, each patient also received written and verbal explanations and instructions from clinic staff. Patients previously completing a questionnaire at any location were excluded from repeat sampling.

A total of 1544 patients returned the form. Eight hundred forty six were seen in one of the two general OB/GYN clinics. The base hospital clinic served 455 of these and 391 were seen in a satellite clinic. Of the patients seen in general clinics, 838 answered the question about satisfaction with services.

The dependent variable, patient satisfaction, was measured by asking "Please indicate your overall satisfaction with OB/GYN care received at the Naval Medical Center San Diego. (NTC Clinic included). Possible answers were Not satisfied...would seek care elsewhere if possible, Satisfied, or Very satisfied. The first two answers were combined to form a variable that measured very satisfied versus not very satisfied.

Predictor variables included age, marital status, duty status (active versus retired), relation (service member versus family member), race (non-Hispanic white, Hispanic, Asian, black, or other/missing), rank and clinic location (main hospital vs satellite clinic). Age and duty status were strongly related. Since duty status had a stronger independent relationship with satisfaction, age was dropped from the multivariate analysis. The lower ranks are indicated by 'E' for enlisted, with E1 being the lowest. Officers are higher ranking and they are indicated by an 'O'. Warrant officers are between enlisted and officer ranks.

Univariate associations between being very satisfied and the predictor variables were tested using chi-square. Multiple logistic regression analysis was used to test the unconditional relationship of each independent variable with satisfaction.

## Results

Over half (56.1 percent) of the users of the general OB/GYN clinics were very satisfied with the services they received (see Table 1). Race, relation and marital status were not significantly related to percent very satisfied. Retired respondents were more likely to be very satisfied than active duty respondents (68.5 percent vs 51.6 percent, p = .0002). Percent very satisfied increased with rank (p = .0002). Women seen at the satellite facility were more likely to report being very satisfied than those seen at the base hospital clinic (62.6 percent vs 49.1 percent, p = .0005).

**Table 1:** Descriptive statistics comparing satisfied (yes) vs. non-satisfied (no) patients

	Yes	No	P
	N (%)	N (%)	
Race			.1787
Non-Hispanic white	212 (57.6)	156 (42.6)	
Asian	40 (49.4)	41 (50.6)	
Black	42 (48.3)	45 (51.7)	
Hispanic	57 (64.0)	32 (36.0)	
Other or missing	32 (55.2)	26 (44.8)	
Duty status			.0002
Active	263 (51.6)	247 (48.4)	
Retired	111 (68.5)	51 (31.5)	
Marital status			.3246
Divorced/separated	22 (68.8)	10 (31.3)	
Married	313 (55.3)	253 (44.7)	
Single	45 (57.0)	34 (43.0)	
Rank			.0002
E1–E4	84 (48.0)	91 (52.0)	
E5–E6	121 (51.9)	112 (48.1)	
E7–E9	59 (58.4)	42 (41.6)	
Warrant-O3	30 (54.5)	25 (45.5)	
O4 and above	75 (76.5)	23 (23.5)	
Missing	14 (66,7)	7 (33.3)	
Relation			.4727
Self	99 (53.8)	85 (46.2)	
Wife or daughter	281 (56.9)	213 (43.1)	
Location			.0005
Main hospital	162 (49.1)	168 (50.9)	
Satellite	221 (62.6)	132 (37.4)	

* number of cases

** significance level

Multivariate analysis of all general clinic patients (Table 2) revealed that retired personnel had an increased odds of being very satisfied compared to active duty respondents (adjusted odds ratio = 1.65). Race was not significant. However, the highest ranking respondents (or women sponsored by high ranking persons) had more than double the odds of being very satisfied than persons of the lowest ranks (AOR = 2.71). Being seen at the satellite clinic independently increased the odds of being satisfied to 1.5 in comparison to being seen in the base hospital clinic.

**Table 2:** Multiple Logistic Regression analysis of Very Satisfied vs Not Very Satisfied (Adjusted Odds Ratios and Confidence Intervals)

	All general patients		Base Hospital		Satellite clinic	
N	672		391		347	
	OR (Conf Interv)	p	OR (Conf Interv)	p	OR (Conf Interv)	p
						
Duty status						
Active	1.0		1.0		1.0	
Retired	1.645 (1.07–2.54)	.025	1.73 (0.80–3.76)	.166	1.59 (0.93–2.71)	.091
Race/ethnicity						
Non-Hispanic White	1.0		1.0		1.0	
Asian	0.77 (0.47–1.28)	.318	0.33 (0.14–0.74)	.006	1.56 (0.77–3.30)	.205
Black	0.80 (0.49–1.30)	.358	0.61 (0.30–1.21)	.155	1.05 (0.52–2.14)	.884
Hispanic	1.64 (0.99–2.72)	.056	1.48 (0.76–2.90)	.251	1.79 (0.83–3.88)	.139
Other/ missing	0.98 (0.55–1.75)	.955	1.09 (0.47–2.52)	.840		
Rank						
E1–E4	1.0		1.0		1.0	
E5–E6	1.06 (0.70–1.59)	.787	1.20 (0.69–2.07)	.524	0.95 (0.50–1.81)	.881
E7–E9	1.16 (0.65–2.04)	.618	1.48 (0.60–3.65)	.395	0.98 (0.45–2.15)	.963
Warrant-O3	1.20 (0.64–2.25)	.577	1.57 (0.66–3.72)	.310	0.84 (0.33–2.17)	.723
O4 and above	2.71 (1.49–4.90)	.001	2.67 (1.11–6.45)	.029	2.76 (1.19–.39)	.018
Missing	0.94 (0.29–3.04)	.923	1.41 (0.27–7.36)	.681	0.56 (.10–3.14)	.509
Location						
Base hospital	1.0					
Satellite	1.49 (1.07–2.06)	.017				

Stratification of the data produced additional insights. When the patients seen in the base hospital were analyzed separately, rank remained important but duty status was no longer significant. Asian respondents had significantly lower odds of being very satisfied relative to non-Hispanic white respondents (AOR = .33, p = .0077) in this subset of the data. Separate analysis of the patients seen in the satellite clinic produced only one significant predictor: persons in the highest rank group had an adjusted odds of 2.76 relative to the lowest rank (p = .0181)

## Discussion

An increasing number of research reports that address patient satisfaction is appearing in the OB/GYN literature. These studies do not employ standard methods for either measurement or analysis and study designs are varied as well.

A critical issue has to do with the measurement of satisfaction. Some studies use mean satisfaction scores [9,10] while others divide subjects into very satisfied versus not very satisfied, as we did [8]. Skewed distributions are the norm in patient satisfaction surveys, due to reluctance on the part of patients to criticize their health care providers, so satisfaction scales usually are dichotomized and analyzed using logistic regression analysis.

Our study differs from many in that it has a large sample size and measures satisfaction using a single item that was dichotomized into satisfied versus not satisfied. It adds to the OB/GYN literature by showing that, as expected, Asian patients are less likely to be very satisfied with care received. This is consistent with findings relating to other patient groups [14-16]. However, we add the proviso that Asian patients are less likely to be satisfied in the base hospital clinic, but not in the satellite clinic. We do not know why this is the case. Approximately the same numbers of Asian patients attended both clinics (43/353 in the satellite and 38/330 in the hospital clinic). Further investigation of this issue is needed so that disparities can be eliminated and so that we can learn of any particular clinic characteristics that Asian patients especially appreciate or dislike.

The second interesting finding in this study is the importance of rank. If the OB/GYN care was being provided by private sector clinics, we might assume that higher ranking officers have higher incomes and thus would receive more attention in a system that is driven by profit. However, the military hospital does not bill patients and so had no direct financial incentive to give special treatment to the 98 higher ranker officers (or spouses) despite their higher incomes. We speculate that rank is important simply because it denotes higher social class and, perhaps, political influence.

Finally, our discovery that the satellite clinic has higher satisfaction levels than the base hospital clinic is worthy of note. We take as a matter of course that managers should monitor satisfaction levels at particular clinics so as to assure that local performance does not drop below norms. Friendliness, patient centered styles of communication [19], shorter wait times, midlevel providers [5,17], seeing the same physician [20], female physicians [21], and racially-concordant physicians [19] may be more common at the satellite clinic while language barriers may be less common; the hospital clinic could take steps to improve these aspects of their services. The base hospital outpatient clinic suffers from some particular disadvantages, including parking problems and a more hectic atmosphere. Further decentralization of OB/GYN services is worthy of consideration as a strategy for addressing these issues. A national study of veteran patients found that veterans seen in free-standing "community-based" outpatient clinics were more satisfied than those seen in traditional hospital outpatient clinics, though the effect was not strong [1]. Easier parking, less travel time and shorter wait times may be at work to produce this result. A study of patients in the Israel Defense Forces found an inverse relationship between satisfaction and clinic size [22]. An observational study of 60 general practices in England found that satisfaction with access to care was better in small practices. Scores for overall satisfaction, continuity of care, and access to care were higher in practices were staff reported a better team climate [23]. If smaller clinics are better able to develop a team spirit, this could explain higher satisfaction levels. Additional research into the relationships between clinic size, team practice, staff morale, and patient satisfaction are needed.

## Conclusion

The results of this study should be treated with caution. The sample may not have been representative of the clinic population. Some cases were dropped from the multivariate analysis due to missing data on one or more variables. Furthermore, San Diego's Naval Medical Center may not be typical of all Naval Medical Centers. Certainly, findings drawn from military clinics may not be generalizable to civilian settings. And, of course, conclusions about patient satisfaction do not necessarily apply to quality of care, since satisfaction reflects lay judgments about quality and may not be accurate on technical matters.

Despite these limitations, we believe that our results contribute to the OB/GYN literature and have practical implications. Clinic managers and physicians should seek to enhance patient satisfaction, especially for patients seen in larger clinic settings and patients in the lower ranks. The reasons why Asian American patients might be less satisfied than other patients should be explored. The mounting evidence in favor of smaller clinics suggests that it is time for further decentralization of services. The purpose behind these suggestions is to make care more personalized and patient-centered.

Our findings suggest a need for additional research. Knowing that Asian patients may be less satisfied begs for further investigation. Lower ratings from Asian Americans may reflect different response tendencies rather than less satisfaction with care. In addition, learning that a satellite clinic produces more satisfied patients suggests that a larger study should be conducted comparing satellites to base hospital clinics.

## Competing interests

The author(s) declare that they have no competing interests.

## Authors' contributions

JR analyzed the data and wrote the first draft of the paper. JL conceived the survey and planned it. SG organized data collection and data entry and critiqued the paper.

## Pre-publication history

The pre-publication history for this paper can be accessed here:


